# Adverse health consequences in COPD patients with rapid decline in FEV_1 _- evidence from the UPLIFT trial

**DOI:** 10.1186/1465-9921-12-129

**Published:** 2011-09-28

**Authors:** Steven Kesten, Bartolome Celli, Marc Decramer, Dacheng Liu, Donald Tashkin

**Affiliations:** 1 Boehringer Ingelheim Pharmaceuticals, Ridgefield, Connecticut, USA; 2 Brigham and Women's Hospital, Boston, Massachusetts, USA; 3 University of Leuven, Leuven, Belgium; 4 David Geffen School of Medicine UCLA, Los Angeles, California, USA

**Keywords:** chronic obstructive pulmonary disease, natural history, forced expiratory volume in 1 second, tiotropium, health-related quality of life

## Abstract

**Background:**

The rate of decline in forced expiratory volume in 1 second (FEV_1_) is representative of the natural history of COPD. Sparse information exists regarding the associations between the magnitude of annualised loss of FEV_1 _with other endpoints.

**Methods:**

Retrospective analysis of UPLIFT^® ^trial (four-year, randomized, double-blind, placebo-controlled trial of tiotropium 18 μg daily in chronic obstructive pulmonary disease [COPD], n = 5993). Decline of FEV_1 _was analysed with random co-efficient regression. Patients were categorised according to quartiles based on the rate of decline (RoD) in post-bronchodilator FEV_1. _The St George's Respiratory Questionnaire (SGRQ) total score, exacerbations and mortality were assessed within each quartile.

**Results:**

Mean (standard error [SE]) post-bronchodilator FEV_1 _increased in the first quartile (Q1) by 37 (1) mL/year. The other quartiles showed annualised declines in FEV_1 _(mL/year) as follows: Q2 = 24 (1), Q3 = 59 (1) and Q4 = 125 (2). Age, gender, respiratory medication use at baseline and SGRQ did not distinguish groups. The patient subgroup with the largest RoD had less severe lung disease at baseline and contained a higher proportion of current smokers. The percentage of patients with ≥ 1 exacerbation showed a minimal difference from the lowest to the largest RoD, but exacerbation rates increased with increasing RoD. The highest proportion of patients with ≥ 1 hospitalised exacerbation was in Q4 (Q1 = 19.5% [tiotropium], 26% [control]; Q4 = 33.8% [tiotropium] and 33.1% [control]). Time to first exacerbation and hospitalised exacerbation was shorter with increasing RoD. Rate of decline in SGRQ increased in direct proportion to each quartile. The group with the largest RoD had the highest mortality.

**Conclusion:**

Patients can be grouped into different RoD quartiles with the observation of different clinical outcomes indicating that specific (or more aggressive) approaches to management may be needed.

**Trial Registration:**

ClinicalTrials.gov number, NCT00144339

## Background

An accelerated loss of lung function relative to healthy individuals is a characteristic feature of chronic obstructive pulmonary disease (COPD) and has been used to define the natural history of the disease [1-3]. The seminal publication by Charles Fletcher and Richard Peto described a rate of loss of forced expiratory volume in 1 second (FEV_1_) in patients with airflow obstruction ranging from 37 ± 8 mL/year in ex-smokers to 80 ± 6 mL/year in heavy smokers (> 15 cigarettes per day), with an overall effect of 64 ± 3 mL/year [2]. Of note, the data are based on a relatively small cohort (n = 792) followed in the 1960s (albeit over a relatively long time interval of 8 years), the population was restricted to men and there is no mention of medication washout or bronchodilator administration preceding spirometry. In addition, the authors describe a 'horse-racing' effect in which there is an inverse relationship to the degree of airflow limitation at baseline and rate of subsequent loss of FEV_1_, the ultimate consequence of which, if left unabated, would be disability and premature death. The 'Fletcher-Peto curve', illustrating the rate of loss over time, has been widely discussed and displayed in peer-reviewed publications. However, the associations of differing magnitudes of loss over time with health outcomes have not been thoroughly documented. Furthermore, inferences from several decades ago may not be currently applicable given the substantial changes that have occurred in the diagnosis and management of COPD [1].

The Understanding Potential Long-term Impacts on Function with Tiotropium (UPLIFT^®^) trial is a four-year randomised, double-blind, placebo-controlled trial of tiotropium 18 μg daily in COPD patients who were permitted to use all respiratory medications throughout the trial other than inhaled anticholinergics [4]. The co-primary outcomes were the rate of decline in pre- and post-bronchodilator FEV_1_. We have therefore conducted a *post-hoc *analysis of the UPLIFT^® ^data and have attempted to categorise patients according to differing rates of decline in post-bronchodilator FEV_1 _to document associations with clinically important health outcomes (i.e. exacerbations and health-related quality of life [HRQoL]).

## Methods

### Study Design

The study design of the UPLIFT^® ^trial has been previously published [4,5]. In brief, UPLIFT^® ^was a four-year, international, randomised, double-blind, placebo-controlled, clinical trial that evaluated the efficacy and safety of tiotropium 18 μg daily administered via HandiHaler^® ^(Boehringer Ingelheim GmbH, Ingelheim, Germany) in the treatment of COPD. All patients in the tiotropium and placebo (control) groups were permitted to use all respiratory medications other than inhaled anticholinergics, as prescribed, throughout the trial. A two- to four-week run-in period was followed by the randomisation visit, a visit at four weeks, and then clinic visits every 12 weeks for the duration of the trial. A washout visit in which patients were asked to administer ipratropium 40 μg four times daily was scheduled for 30 days following completion of randomised study drug.

The study was approved by ethics committees and institutional review boards and was conducted in accordance with the Helsinki Declaration. Written informed consent was obtained from all patients.

### Study Population

Men and women who were aged ≥ 40 years, had ≥ 10 pack-year history of smoking and had evidence of airflow limitation (defined by a post-bronchodilator FEV_1 _≤ 70% predicted and an FEV_1_/forced vital capacity (FVC) ≤ 0.70) were included. Exclusion criteria included a history of asthma, pulmonary resection, unstable disease, recent myocardial infarction and recent hospitalisation for congestive heart failure. Other criteria are outlined in previous publications and were designed to exclude patients who might not reasonably be assumed to be able to complete the trial or had an underlying disease that would interfere with the interpretation of the study results [4,5].

### Procedures

Spirometry was performed according to American Thoracic Society criteria at baseline, four weeks and every six months following randomisation [6]. After screening, spirometry was performed prior to, and following, short-acting bronchodilator and study drug administration. Short-acting bronchodilators were administered sequentially (ipratropium bromide four actuations, albuterol four actuations 60 minutes later) with spirometry being performed 30 minutes following the last dose. HRQoL was measured using the St George's Respiratory Questionnaire (SGRQ) at baseline and every six months [7]. Exacerbations were recorded on case report forms and were required to follow a standard definition as follows: an increase in or the new onset of more than one respiratory symptom (cough, sputum, sputum purulence, wheezing or dyspnea) lasting three days or more and requiring treatment with an antibiotic or a systemic corticosteroid. All adverse events were recorded throughout the period of study drug administration. In addition, investigators were requested to collect vital status information on patients who prematurely discontinued study medication during the four years of the trial.

### Data Analysis

Spirometric variables (FEV_1_, FVC and slow vital capacity) and SGRQ were analysed using random-effects models. The decline of lung function over time was analysed with random co-efficient regression. Patients were categorised according to quartiles based on the annualised rate of post-bronchodilator FEV_1 _decline. As treatment intervention with tiotropium would potentially interact with the health outcomes measured, the analysis was stratified by treatment group. Hazard ratios for exacerbations, hospitalised exacerbations and death were determined using Cox regression. The numbers of exacerbations and associated hospitalisations were calculated using Poisson regression with adjustment for overdispersion and treatment exposure. Baseline data were not included in the FEV_1 _model.

## Results

### Demographics

**Figure 1:**
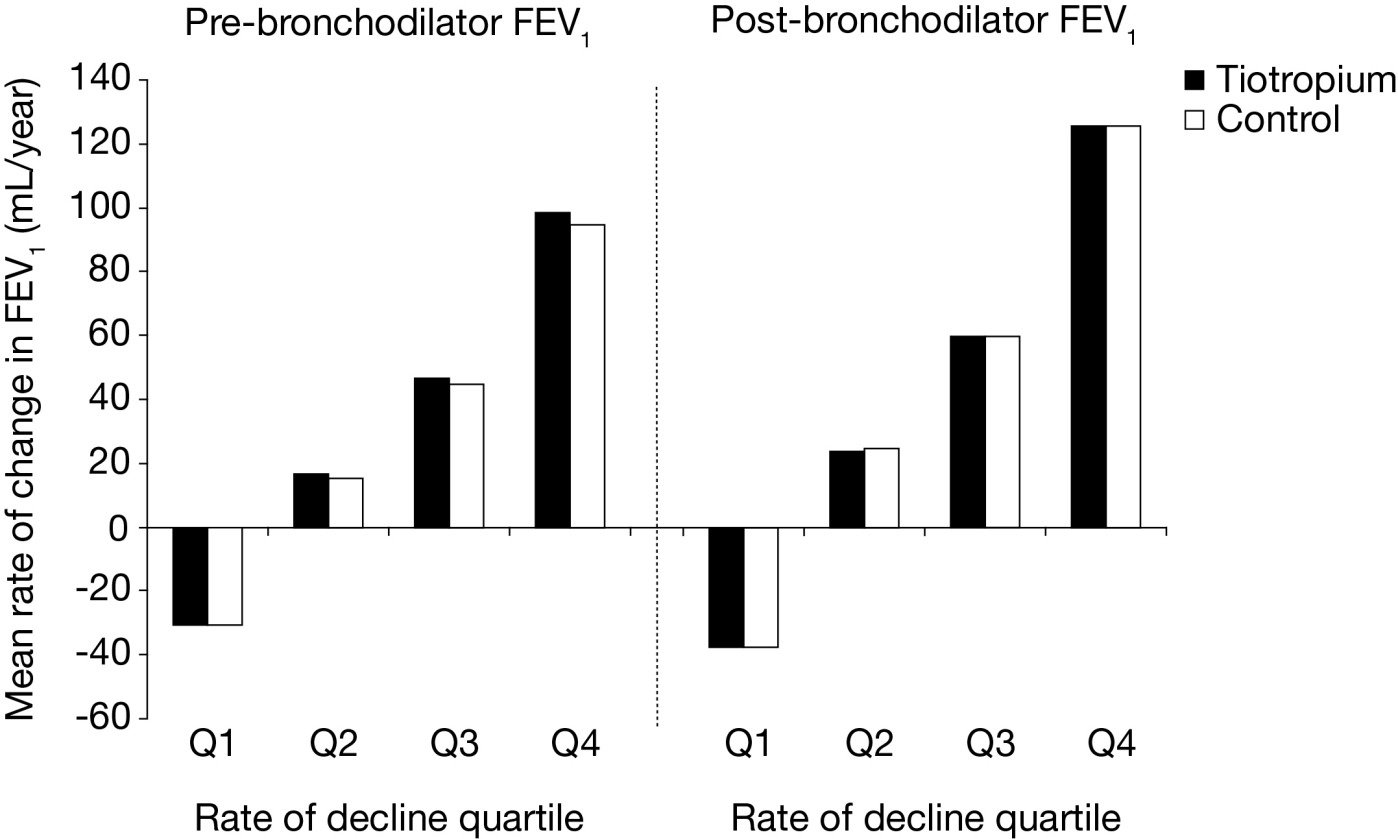
**Annualised RoD in pre- and post-bronchodilator FEV_1 _by quartiles for tiotropium and control groups**. Abbreviations: RoD, rate of decline; FEV_1_, forced expiratory volume in 1 second.

A total of 4964 patients from the total population (n = 5993) had evaluable spirometry for evaluation of rate of decline of FEV_1_. The number of patients within each quartile by treatment group is as follows: Q1 (tiotropium 661, control 580), Q2 (tiotropium 645, control 596), Q3 (tiotropium 627, control 614), Q4 (tiotropium 621, control 620). Overall, the mean (standard error [SE]) post-bronchodilator FEV_1 _increased in the first quartile (Q1) by 37 (1) mL/year (Figure 1). The other quartiles showed annualised mean (SE) declines in FEV_1 _(mL/year) as follows: Q2 = 24 (1), Q3 = 59 (1) and Q4 = 125 (2). The corresponding rates for post-bronchodilator FVC were as follows: increase in Q1 = 41 (4), decreases in Q2, Q3 and Q4 (Q2 = 33 [4], Q3 = 81 [4] and Q4 = 179 [5]). The baseline characteristics of the population by quartiles of rate of decline in post-bronchodilator FEV_1 _are displayed in Table 1. Approximately 75% were men with an average age of 64 years. Overall, age, gender, respiratory medication use at baseline and SGRQ total score did not appear to distinguish groups, although the subjects in the highest quartile tended to be younger and more predominantly male. The largest rate of decline included the highest proportion of current smokers. Patients with the largest rate of decline had less severe lung disease on average at baseline as seen by FEV_1_, FVC and Global Initiative for Chronic Obstructive Lung Disease (GOLD) stage (Table 2).

**Table 1:** Baseline characteristics of the UPLIFT^® ^study population according to quartiles of RoD in post-bronchodilator FEV_1_

	Rate of decline quartile			
	
	Q1 (n = 1241)	Q2 (n = 1241)	Q3 (n = 1241)	Q4 (n = 1241)
**Male (%)**	77	72	75	80

**Age (years)**	65 ± 9	65 ± 8	64 ± 8	63 ± 9

**Smoking history (%)**				
**Ex-smoker**	73	76	71	63
**Current smoker**	27	24	29	37

**Duration of COPD (years)**	10 ± 8	10 ± 8	10 ± 8	9 ± 7

**Inhaled respiratory drugs (%)**				
**LABA**	61	60	62	58
**ICS**	63	61	60	61
**LABA + ICS**	50	48	49	47
**Anticholinergic**	43	44	43	46

**SGRQ total score (units)**	45 ± 18	45 ± 17	45 ± 17	46 ± 17

Abbreviations: UPLIFT^®^, Understanding Potential Long-Term Impacts on Function with Tiotropium; RoD, rate of decline; FEV_1_, forced expiratory volume in 1 second; COPD, chronic obstructive pulmonary disease; LABA, long-acting β_2 _agonist; ICS, inhaled corticosteroids; SGRQ, St George's Respiratory Questionnaire.

**Table 2:** Baseline spirometry of the UPLIFT^® ^study population according to quartiles of RoD in post-bronchodilator FEV_1_.

	Rate of decline quartile			
	
	Q1 (n = 1241)	Q2 (n = 1241)	Q3 (n = 1241)	Q4 (n = 1241)
**FEV_1 _(L)**				
**Pre-bronchodilator**	1.11 ± 0.40	1.06 ± 0.40	1.09 ± 0.39	1.21 ± 0.39
**Post-bronchodilator**	1.33 ± 0.44	1.28 ± 0.44	1.32 ± 0.44	1.47 ± 0.42

**FEV_1 _(% predicted)**				
**Pre-bronchodilator**	39.7 ± 12.1	39.1 ± 12.0	39.3 ± 12.1	41.4 ± 11.3
**Post-bronchodilator**	47.6 ± 12.7	47.3 ± 12.8	47.8 ± 12.6	50.0 ± 11.5

FEV_1 _(% increase)*	22.6 ± 18.7	23.7 ± 17.4	24.1 ± 17.9	23.2 ± 18.2

**FVC (L)**				
**Pre-bronchodilator**	2.56 ± 0.81	2.51 ± 0.81	2.62 ± 0.79	2.92 ± 0.79
**Post-bronchodilator**	2.99 ± 0.87	2.97 ± 0.86	3.10 ± 0.83	3.42 ± 0.86

**FVC (% predicted)**				
**Pre-bronchodilator**	72.7 ± 17.6	73.6 ± 17.6	75.2 ± 18.1	79.0 ± 17.3
**Post-bronchodilator**	84.9 ± 18.7	87.2 ± 18.3	89.1 ± 19.1	92.7 ± 17.9

**GOLD stage (%)**				
**II**	45	46	45	54
**III**	44	43	45	40
**IV**	9	10	8	4

*Increase from baseline following ipratropium four actuations and albuterol four actuations.

Values displayed as means ± SD unless otherwise indicated.

Abbreviations: UPLIFT^®^, Understanding Potential Long-Term Impacts on Function with Tiotropium; RoD, rate of decline; FEV_1_, forced expiratory volume in 1 second; FVC, forced vital capacity; GOLD, Global Initiative for Chronic Obstructive Lung Disease; SD, standard deviation.

### Health Outcomes

**Figure 2:**
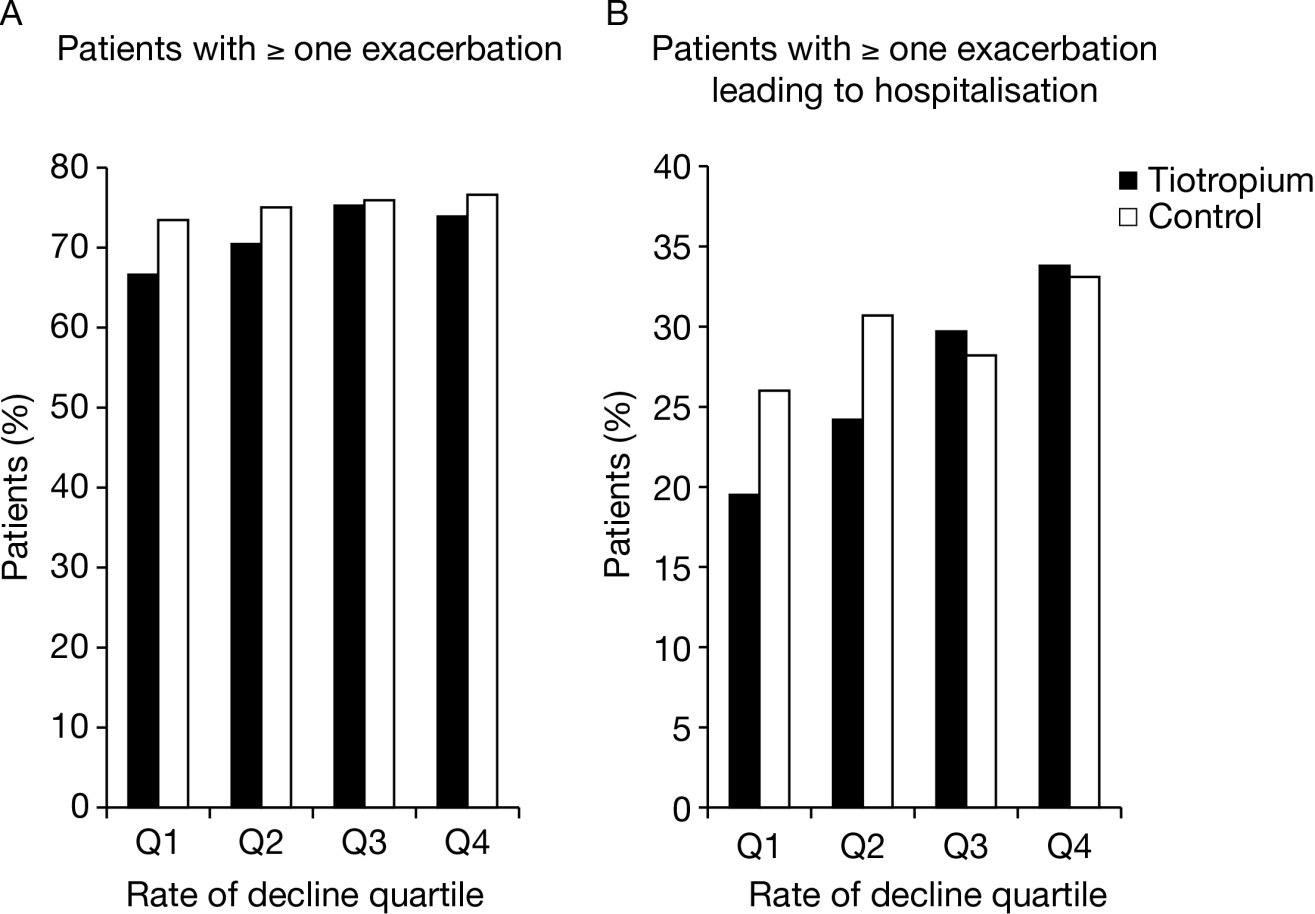
**Incidence of exacerbations according to quartiles of post-bronchodilator FEV_1 _RoD**. Proportion of patients with (A) at least one exacerbation and (B) at least one hospitalised exacerbation. Abbreviations: FEV_1_, forced expiratory volume in 1 second; RoD, rate of decline.

The proportion of patients with at least one exacerbation over four years showed a minimal difference from the lowest rate of decline (Q1 = 66.6% [tiotropium] and 73.3% [control]) to the largest rate of decline (Q4 = 73.8% [tiotropium] and 76.6% [control]) (Figure 2A). However, in general, there was an increased rate of exacerbations with increasing rate of decline in post-bronchodilator FEV_1 _(Table 3). The highest proportion of patients with at least one hospitalised exacerbation was in the group with the largest rate of decline (Q1 = 19.5% [tiotropium] and 26% [control], Q4 = 33.8% [tiotropium] and 33.1% [control]) (Figure 2B). Overall, the time to the first exacerbation and first hospitalised exacerbation was shorter with increasing rate of decline in FEV_1 _quartiles, although results were somewhat inconsistent, especially for patients in the third quartile in the control group (Table 4).

**Table 3:** Number of exacerbations per patient-year according to post-bronchodilator FEV_1 _RoD quartiles.

	Tiotropium	Control	Rate ratio (tiotropium/control)* (95% CI)
Q1	0.63	0.80	0.79 (0.69 to 0.91)

Q2	0.64	0.83	0.77 (0.68 to 0.88)

Q3	0.75	0.78	0.96 (0.85 to 1.09)

Q4	0.85	0.91	0.93 (0.82 to 1.06)

*Rate ratio based on Poisson regression with adjustment for overdispersion.

Abbreviations: FEV_1_, forced expiratory volume in 1 second; RoD, rate of decline; CI, confidence interval.

**Table 4:** Time to first exacerbation and first hospitalised exacerbation according to post-bronchodilator FEV_1 _RoD quartiles.

	Tiotropium	Control	Hazard ratio(tiotropium versus control)*(95% CI)
**Time to first exacerbation, median month**			

Q1	19.4	14.6	0.80 (0.70 to 0.92)
Q2	22.4	13.6	0.83 (0.73 to 0.94)
Q3	16.6	16.3	0.97 (0.86 to 1.11)
Q4	14.9	12.7	0.88 (0.78 to 1.01)

**Time to first exacerbation leading to hospitalization (first quartile), month**			

Q1	NA	39.9	0.72 (0.57 to 0.91)
Q2	47.2	32.5	0.73 (0.59 to 0.90)
Q3	37.0	40.2	1.05 (0.86 to 1.30)
Q4	28.5	26.0	0.98 (0.81 to 1.18)

*Hazard ratio based on Cox regression with treatment, rate of decline quartile, and rate of decline by treatment interaction as covariates.

Abbreviations: FEV_1_, forced expiratory volume in 1 second; RoD, rate of decline; CI, confidence interval.

**Figure 3:**
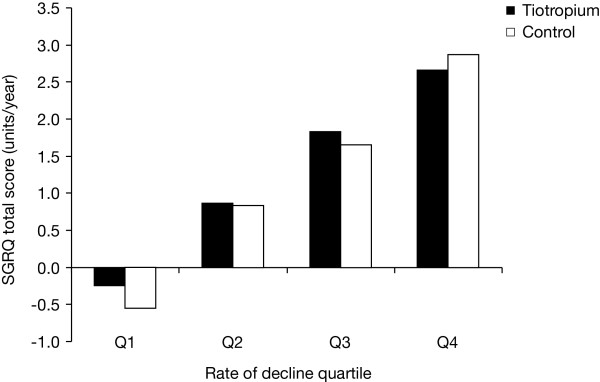
**Annualised decline in SGRQ total score (units/year) according to post-bronchodilator FEV_1 _RoD quartiles**. Abbreviations: SGRQ, St George's Respiratory Questionnaire; FEV_1_, forced expiratory volume in 1 second; RoD, rate of decline.

**Figure 4:**
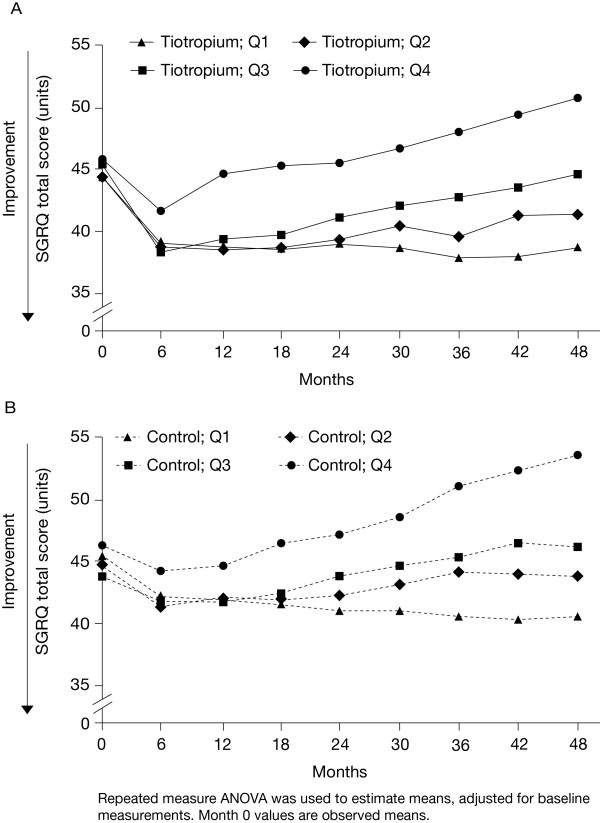
**SGRQ total score over four years by annualised RoD in post-bronchodilator FEV_1_**. (A) tiotropium and (B) control groups. Abbreviations: SGRQ, St George's Respiratory Questionnaire; RoD, rate of decline; FEV_1_, forced expiratory volume in 1 second; ANOVA, analysis of variance.

The annualised rate of decline in the SGRQ total score increased linearly with each quartile increase in the post-bronchodilator decline in FEV_1 _(Figure 3). The range was from an improvement over time in Q1 (control 0.55 [0.19] units, tiotropium 0.24 [0.17] units) to the largest worsening in the Q4 group (control 2.87 [0.19] units, tiotropium 2.66 [0.18] units). The pattern of treatment response throughout the trial is illustrated in Figure 4.

### Mortality

**Figure 5:**
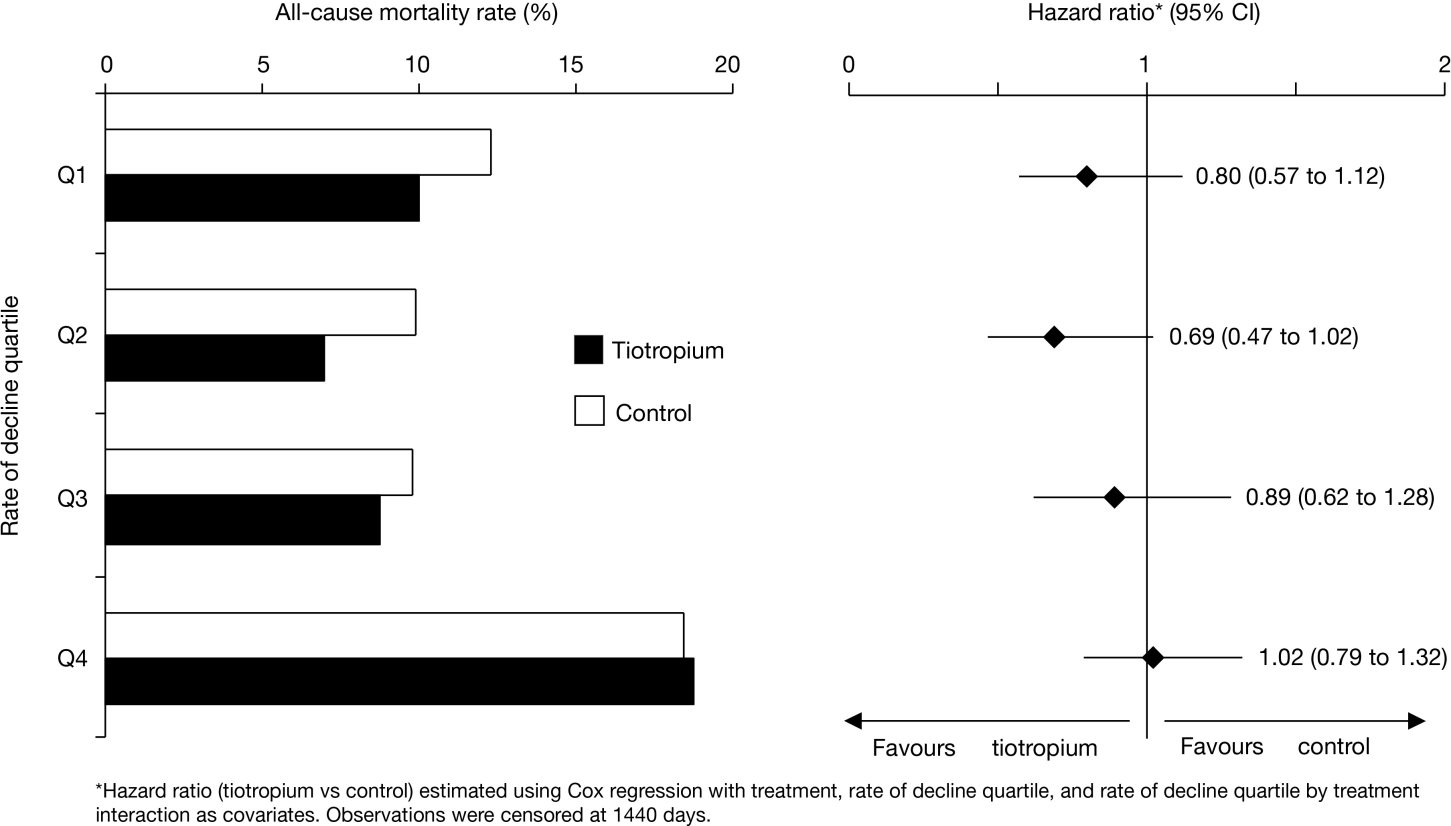
**Proportion of patients who died during treatment according to post-bronchodilator FEV_1 _RoD quartiles**. Protocol defined treatment period (1440 days). Data also include those patients who discontinued study drug. Abbreviations: FEV_1_, forced expiratory volume in 1 second; CI, confidence interval; RoD, rate of decline.

There appeared to be no distinguishing pattern of risk of a fatal event among the first three quartiles (Q1, Q2, Q3); however, the group with the largest rate of FEV_1 _decline (Q4) had the highest overall mortality rate (Figure 5). For all of the hazard ratios, the 95% confidence intervals included one.

## Discussion

The four-year UPLIFT^® ^trial provided data that permitted examination of associations of the magnitude of lung function decline with changes in the clinically relevant health outcomes of exacerbations, HRQoL and survival [4,5]. In order to examine patterns, the annualised rate of loss of post-bronchodilator FEV_1 _was divided into quartiles. Other than smoking status, demographics and respiratory medication use appeared to be largely similar among groups; and the most prominent difference at baseline was lung function, which appeared to be least impaired in those with the most rapid loss in FEV_1_. In longitudinal analyses, the patterns associated with quartiles of increasing rate of decline in FEV_1 _indicated a relationship to an accelerated loss of HRQoL, increased rate of exacerbations, increased risk of a hospitalised exacerbation and increased risk of death.

COPD is described as a disease of progressive airflow limitation, for which the most described marker remains the serial measurement of FEV_1 _[1]. The publication by Fletcher and Peto placed FEV_1 _decline over time at the forefront of biomarkers that illustrate disease progression [2]. However, the utility of any biomarker is somewhat dependent on its predictive value or associations with other clinically important endpoints. In this regard, there are two main analytic approaches to FEV_1_, regardless of whether it is described as an absolute volume or as a percentage of predicted normal. The first approach is the static single-point measurement. While there is a clear relationship of FEV_1 _severity to subsequent mortality, the descriptions of which date back several decades [8,9], composite measures such as the BODE index that have FEV_1 _as a part of the measure appear to have higher predictive values [10]. Additionally, other parameters such as comorbidities and serum markers should continue to be explored for associations with disease progression. Yet a single measurement of FEV_1 _is poorly associated with other health outcomes such as exercise tolerance and quality of life. This may relate to issues of a single measurement in a disease that is known to have substantial day-to-day or week-to-week variability. The second analytic approach is serial measurements over time examining the pattern of change (i.e. slope).

Most studies have described FEV_1 _decline in the context of the effects of cigarette smoking and cessation of exposure to cigarette smoke with minimal data being published on the associations of rate of decline in FEV_1 _with other health outcomes [3,11]. The rate of loss of lung function over time was recently described in the Towards a Revolution in COPD Health (TORCH) trial [12]. The authors noted that an increased rate of decline was associated with more frequent exacerbations [12]. A rapid decline in FEV_1 _has been associated with more frequent exacerbations of COPD in other studies [13,14] and severe exacerbations have been shown to be associated with premature death [15]. The remaining studies have used FEV_1 _as an outcome measure but have not described changes in FEV_1 _over time relative to changes over time in other outcomes [16-19].

Our current population showed that age and gender did not differ significantly among the quartiles of FEV_1 _decline. Indeed, it appears that physician's treatment patterns, as viewed by concomitant respiratory medication prescription, also did not distinguish patient groups, at least as defined by disease severity at baseline (GOLD stage). Continued smoking did show differences, which is entirely expected and also provides evidence that the subgroups are valid [20]. The most rapid loss was observed in patients who appeared to have somewhat milder disease, either by absolute FEV_1_, FEV_1 _percent predicted or FVC. This seems to be somewhat contrary to the 'horse-racing' effect described by Fletcher and Peto, but may highlight the need for earlier and aggressive intervention in patients with COPD [2]. There may be several possible explanations for the differences in our study and that of Fletcher and Peto as follows: (a) different populations (British coal miners vs. well characterized multinational representation), (b) sample size (larger in the UPLIFT trial), (c) survival (more likely to have severe disease in the UPLIFT trial due to advance in health care), and (d) environmental conditions (coal miners and likely higher levels of particulate exposure vs. current air quality standards). A consistency is seen with recent data that show improvements in outcomes with long-term pharmacotherapy in GOLD stage II patients (UPLIFT^® ^and TORCH), as well as significant benefit in patients not previously receiving maintenance respiratory medication [4,21,22].

Certain limitations should be noted. The calculation of FEV_1 _decline was only continued while the patient received study drug and was not assessed following premature discontinuation of study drug. Premature discontinuation of study drug occurred in more control patients (45%) than tiotropium patients (36%). However, there was still substantial exposure to both study drugs during the trial with multiple measurements over years in most patients in the current analysis. Suissa describes the phenomenon of regression to the mean, which certainly could contribute to our findings; however, the inclusion of a data point closely following randomisation (i.e. at 30 days) should limit this [23]. Each treatment group needs to be considered independently since between-treatment comparisons are not valid, given that subgroups are based on outcomes (i.e. rate of decline in FEV_1_). Furthermore, there is a bias toward the more severe COPD patients preferentially in the tiotropium group continuing to completion, due to the effectiveness of the intervention, which created unequal groups as the trial progressed. Nevertheless, the pattern of association of quartiles to rate of decline in FEV_1 _can be observed within both treatment groups.

The study's strengths included the large sample size, the rather liberal inclusion criteria, the length of the trial and the rigorous nature of the data collection. The latter is particularly true of spirometry, which pre-specified a stringent protocol and involved standardised equipment, study specific software and a centralised review of all measurements.

The current results provide supportive evidence of the relevance of serial measurements in FEV_1 _over time. A rapid loss of lung function appears to identify a group at increased risk for more frequent exacerbations, severe exacerbations (i.e. hospitalised events), an accelerated loss of HRQoL and premature mortality. FEV_1 _measurement is simple, relatively inexpensive, widely available and well standardised with published normative values. COPD is a chronic disease and patients with COPD may be followed clinically for several decades. Identification of rapid decliners could be clinically useful since such patients may represent a unique subset of patients who require aggressive interventions. It is relevant to note that there can be marked heterogeneity between individuals regarding the absolute annual loss of lung function, which will be influenced by their genetic background and environmental factors.

Identification of patients with the most rapid decline in lung function, particularly in the early stages of COPD, would require a study of reproducibility of FEV_1 _and its decline in individual patients, possibly at short intervals (e.g. 3-6 monthly). Before translating to clinical practice, further research is needed regarding the reproducibility and patterns of rate of decline in individual patients. At a minimum, additional research is required to identify factors, other than continued exposure to noxious fumes and particulate matter (i.e. tobacco smoke), such as biomarkers and genetic patterns that can both predict a rapid decline and lead to novel approaches in treating such patients. Another issue to consider is whether rapid decliners represent a distinct subset of COPD. In an editorial, Rennard and Vestbo described an approach in which COPD can be considered an orphan disease requiring unique approaches to different forms of the disease [24]. Moreover, in another editorial, Reilly wrote about COPD being the sum of many small COPDs [25]. Whether this is the case for rapid or slow decliners remains to be determined.

## Conclusion

The current data suggest that patients can be divided into different rates of decline with the observation of different clinical outcomes indicating that specific (or more aggressive) approaches to management are needed.

## List of abbreviations

ANOVA: analysis of variance; CI: confidence interval; COPD: chronic obstructive pulmonary disease; FEV_1 _: forced expiratory volume in 1 second; FVC: forced vital capacity; GOLD: Global Initiative for Chronic Obstructive Lung Disease; HRQoL: health-related quality of life; ICS: inhaled corticosteroids; LABA: long-acting β_2 _agonist; GOLD: Global Initiative for Chronic Obstructive Lung Disease; RoD: rate of decline; SGRQ: St George's Respiratory Questionnaire; SD: standard deviation; SE standard error; TORCH: Towards a Revolution in COPD: Health; UPLIFT^® ^: Understanding Potential Long-Term Impacts on Function with Tiotropium.

## Competing interests

Donald Tashkin has received: honoraria from Boehringer Ingelheim, Pfizer, Dey Labs, AstraZeneca, Teva Pharmaceuticals, GlaxoSmithKline; consultancy fees from Boehringer Ingelheim, Novartis, Dey Labs, Schering-Plough, AstraZeneca; grants from Almirall, AstraZeneca, Boehringer Ingelheim, Chiesi, Dey, GlaxoSmithKline, Novartis, Pfizer, Schering-Plough, Sepracor; and speaker bureau fees from Boehringer Ingelheim, Novartis, Dey Labs, Schering-Plough, and AstraZeneca. Bart Celli has received: honoraria and consultancy fees from Almirall, AstraZeneca, Boehringer Ingelheim, and GlaxoSmithKline; and grants from Boehringer Ingelheim, Forest, and GlaxoSmithKline. Marc Decramer has received: honoraria from Boehringer Ingelheim, Pfizer, and AstraZeneca; consultancy fees from Boehringer Ingelheim and GlaxoSmithKline; grants from AstraZeneca and GlaxoSmithKline; and speaker bureau fees from Boehringer Ingelheim, GlaxoSmithKline, Nycomed, and Dompé. Steven Kesten is a previous employee of Boehringer Ingelheim. Steven Kesten is a current employee of, and holds shares in Uptake Medical Corp. Dacheng Liu is an employee of Boehringer Ingelheim.

## Authors' contributions

Drs MD, BC, DT, and SK (former employee of Boehringer Ingelheim and currently at Uptake Medical, Tustin, California) contributed to the design and conduct of the UPLIFT^® ^trial. Dr L (Boehringer Ingelheim) provided statistical support. All authors contributed to the data analyses, the interpretation of the data and the composition of the manuscript and were involved in the decision to submit the paper for publication.
